# *In Vivo* Behavior of the Tandem Glycine Riboswitch in *Bacillus subtilis*

**DOI:** 10.1128/mBio.01602-17

**Published:** 2017-10-31

**Authors:** Arianne M. Babina, Nicholas E. Lea, Michelle M. Meyer

**Affiliations:** Department of Biology, Boston College, Chestnut Hill, Massachusetts, USA; NICHD

**Keywords:** RNA structure, riboswitch, biofilms, gene regulation, swarming

## Abstract

In many bacterial species, the glycine riboswitch is composed of two homologous ligand-binding domains (aptamers) that each bind glycine and act together to regulate the expression of glycine metabolic and transport genes. While the structure and molecular dynamics of the tandem glycine riboswitch have been the subject of numerous *in vitro* studies, the *in vivo* behavior of the riboswitch remains largely uncharacterized. To examine the proposed models of tandem glycine riboswitch function in a biologically relevant context, we characterized the regulatory activity of mutations to the riboswitch structure in *Bacillus subtilis* using β-galactosidase assays. To assess the impact disruptions to riboswitch function have on cell fitness, we introduced these mutations into the native locus of the tandem glycine riboswitch within the *B. subtilis* genome. Our results indicate that glycine does not need to bind both aptamers for regulation *in vivo* and mutations perturbing riboswitch tertiary structure have the most severe effect on riboswitch function and gene expression. We also find that in *B. subtilis*, the glycine riboswitch-regulated *gcvT* operon is important for glycine detoxification.

## IMPORTANCE

The glycine riboswitch is a unique *cis*-acting mRNA element that contains two tandem homologous glycine-binding domains that act on a single expression platform to regulate gene expression in response to glycine. While many *in vitro* experiments have characterized the tandem architecture of the glycine riboswitch, little work has investigated the behavior of this riboswitch *in vivo*. In this study, we analyzed the proposed models of tandem glycine riboswitch regulation in the context of its native locus within the *Bacillus subtilis* genome and examined how disruptions to glycine riboswitch function impact organismal fitness. Our work offers new insights into riboswitch function *in vivo* and reinforces the potential of riboswitches as novel antimicrobial targets.

## INTRODUCTION

Riboswitches are structured mRNA elements found in the 5′ untranslated regions of bacterial transcripts that regulate gene expression in response to interactions with specific small molecules. They typically consist of a ligand-binding domain, or aptamer, followed by an expression platform that mediates conformational changes occurring upon ligand binding into modulation of transcription termination, translation initiation, RNA degradation, or alternative splicing. Over 20 distinct classes of riboswitches that interact with a diverse array of metabolites and control genes responsible for a number of biologically essential processes have been characterized to date (for a review, see references 1 to 3).

As a result of their association with genes essential for survival or pathogenesis, riboswitches are proposed as novel targets for antimicrobials (4–7). Nevertheless, most riboswitch research has been confined to *in vitro* experimental approaches. Apart from select regulatory assays and drug discovery efforts (4, 5, 8, 9), limited work has been conducted to examine how the results of *in vitro* studies translate to *in vivo* properties of riboswitches or determine how riboswitch function impacts organismal fitness (10).

The glycine riboswitch was among the first riboswitches discovered, and more than 7,000 homologs have subsequently been identified across numerous bacterial species (11, 12). Many examples of the glycine riboswitch are composed of two tandem glycine-binding aptamers, followed by a single expression platform. Currently, the glutamine riboswitch is the only other known tandem riboswitch in which two or more separate homologous metabolite-sensing aptamers are proposed to act together on a single expression platform (13). Other examples of tandem riboswitches consist of two or more complete and functionally independent riboswitches (14–16), and a few riboswitch aptamers that bind two ligand molecules within a single structure have been described (17–19).

Because of its unique architecture, the structure and molecular dynamics of the tandem glycine riboswitch have been the subject of numerous biochemical and biophysical studies (11, 12, 20–30). Initial experiments demonstrated cooperative glycine-binding behavior between the two homologous aptamers *in vitro* (11, 21, 23), and subsequent work proposed a model of sequential glycine binding and asymmetrical cooperativity (20–25). Later studies identified a highly conserved leader-linker kink-turn interaction that promotes riboswitch folding and glycine binding (26–29). These results suggested that the full-length tandem glycine riboswitch did not demonstrate cooperative binding and the observed cooperativity was an artifact of the truncated constructs utilized for *in vitro* characterization. The current model of tandem glycine riboswitch function is based on extensive analysis of the glycine-binding and dimerization affinities of the two aptamers and proposes that aptamer dimerization and ligand binding are linked equilibria; dimerization interactions promote glycine binding, and subsequent glycine binding further stabilizes riboswitch tertiary structure to allow for gene control (30). Furthermore, recent work with naturally occurring “singlet” glycine riboswitches (one aptamer followed by a single expression platform) demonstrated that singlet riboswitches bind glycine with affinities comparable to those with the tandem aptamer architecture. However, singlet glycine riboswitches still require interactions between the aptamer domain and a flanking stem-loop “ghost aptamer” for proper folding and ligand-binding activity (12).

While the *in vitro* techniques applied to the tandem glycine riboswitch provide invaluable insight into structure and mechanism of action, such experiments do not always accurately reflect behavior within the cell. To examine the tandem glycine riboswitch in a more biologically relevant context, we characterized the expression changes resulting from a panel of *Bacillus subtilis* glycine riboswitch mutants designed to probe aspects of the *in vitro* folding models using β-galactosidase reporter assays. To understand the impact such changes have on organismal fitness, we introduced these mutations into the native locus of the tandem glycine riboswitch preceding the *gcvT* glycine cleavage operon within the *B. subtilis* genome and examined organismal phenotype under a variety of conditions.

Our data suggest that mutations disrupting first aptamer tertiary structure have the greatest impact on tandem glycine riboswitch regulation and gene expression. We find that glycine-induced expression of the *gcvT* operon is necessary for *B. subtilis* growth, swarming motility, and biofilm formation in high-glycine environments. However, constitutive expression of the *gcvT* operon in the absence of glycine also has adverse effects on growth, emphasizing the importance of the tandem glycine riboswitch function as a genetic "on" switch in response to excess glycine.

## RESULTS

### Double ligand occupancy is not necessary to elicit a regulatory response.

**FIG 1:**
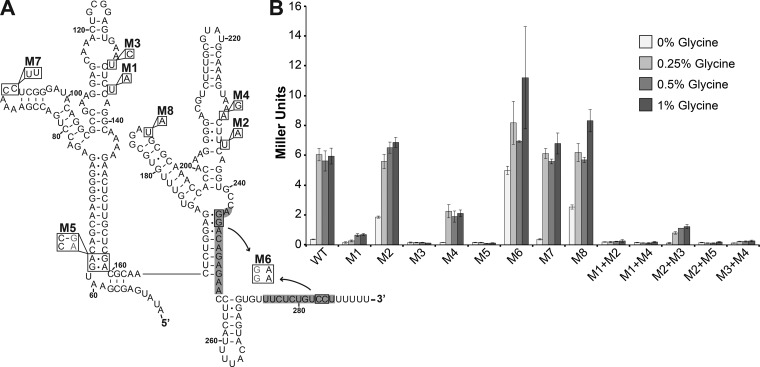
Regulatory activity of glycine riboswitch mutations. (A) Secondary structure of the *B. subtilis* glycine riboswitch with mutations M1 to M8. Nucleotides are numbered from the transcript start site, +1 (59). Gray shading highlights nucleotides that base pair to form the transcription terminator stem when the riboswitch is in the "off" conformation. (B) β-Galactosidase activities of riboswitch mutant constructs in the presence of increasing glycine concentrations. Each value is the mean of three or more independent experimental replicates; error bars represent the standard error of the mean across biological replicates. WT, wild type.

To investigate the impacts of disruptions to glycine binding and interaptamer interactions on glycine riboswitch *in vivo* regulation, we examined a number of mutations to the tandem riboswitch structure in *B. subtilis* using β-galactosidase reporter assays (Fig. 1A). Each mutant riboswitch sequence, including the first nine codons of *gcvT*, was cloned in-frame as a translational fusion with a *lacZ* reporter under the control of the native *gcvT* operon promoter and then stably integrated as a single copy into the *amyE* locus of the *B. subtilis* 168 genome. The regulatory properties of each riboswitch mutant were then assessed by measuring the β-galactosidase activity of reporter strains grown with various glycine concentrations. As expected, the wild-type riboswitch construct behaved as a genetic "on" switch in the presence of glycine (Fig. 1B). The β-galactosidase activity of the wild-type riboswitch reporter strain increased approximately 17-fold upon the addition of 0.25% glycine to the medium, and this activity level was maintained for all subsequent glycine concentrations tested.

Mutations M1 and M2 target the glycine-binding domains on the first and second aptamers, respectively (Fig. 1A). These bases have been shown to be important for glycine binding via inline probing experiments with both the *B. subtilis* and *Vibrio cholerae* riboswitches and are in close proximity to the ligand in the crystal structure of the *Fusobacterium nucleatum* RNA (11, 21, 23, 25). Additionally, previous *in vitro* work with homologous *V. cholerae* glycine riboswitch mutants demonstrated that each binding-site mutation disrupts glycine binding independently and that the ability of one aptamer to bind glycine only has a small effect on the glycine-binding affinity of the other aptamer (30).

Mutation M1 significantly reduced but did not completely abolish riboswitch responsiveness to glycine and downstream gene expression. The maximum activities measured for the M1 construct were approximately one-tenth of those obtained with the wild-type construct (0.7 ± 0.06 versus 5.9 ± 0.52 Miller units, respectively, in 1% glycine). Surprisingly, the M2 mutation to the glycine-binding pocket on the second aptamer did not affect riboswitch regulation in response to glycine or maximum gene expression levels (6.9 ± 0.33 versus 5.9 ± 0.52 Miller units from the wild type in 1% glycine) (Fig. 1B).

This finding is in contrast to previous *in vitro* studies with the *V. cholerae* riboswitch demonstrating that disrupting the glycine-binding site on the second aptamer has the greatest impact on the binding affinity of the first aptamer (30). The M2 single mutation also resulted in higher basal β-galactosidase activity than that obtained with the wild-type construct at 0% glycine. The proximity of the M2 mutation to the adjacent terminator stem may affect terminator stability, resulting in the elevated basal constitutive expression. In agreement with previously published findings, the M1+M2 double glycine-binding mutant construct abrogated regulation (30). On the basis of the behavior of the M1 and M2 single and double mutant constructs, glycine must bind to at least one aptamer to promote a regulatory response and glycine binding to the first aptamer is necessary to drive maximum downstream gene expression.

### Interaptamer interactions are important for tandem glycine riboswitch regulation.

The M3 and M4 mutations were designed on the basis of structural data for the homologous *F. nucleatum* aptamers and target the U-A γ interaptamer contacts that are proposed to play a crucial role in communicating the status of glycine binding between the two aptamers (Fig. 1A) (25). Mutating this dimerization interface within the first aptamer (M3) resulted in a complete loss of regulation. However, the M4 mutation to the second aptamer retained regulatory activity in response to glycine, although the maximum activity obtained was about one-third of that measured with the wild-type riboswitch construct (Fig. 1B).

This finding is the reverse of what has been reported for previous *in vitro* mutational analyses of the dimerization interface. A point mutation homologous to M4 in the *V. cholerae* riboswitch was found to have the most severe impact on aptamer dimerization via *trans* gel shift assays (30). Mutating the γ dimerization interface on both aptamers (M3+M4) abrogated glycine riboswitch function and yielded basal expression levels that were slightly higher than those measured for the M3 single mutant construct (0.27 ± 0.06 Miller units for the M3+M4 construct compared to 0.12 ± 0.02 Miller units for the M3 construct at 1% glycine).

To examine the importance of interaptamer interactions for glycine binding and consequent regulation, we combined each dimerization mutant with the mutation to the glycine-binding pocket on the opposite aptamer (M1+M4 and M2+M3). The M1+M4 mutant displayed total loss of regulation and downstream expression, whereas the M2+M3 double mutant retained regulatory activity and modest downstream expression.

Similar to the single dimerization mutants (M3, M4), the behavior of the M1+M4 and M2+M3 double mutant constructs was the opposite of what has been previously observed. Equilibrium dialysis assays with a *V. cholerae* glycine riboswitch construct homologous to our M2+M3 construct resulted in the greatest reduction in glycine-binding affinity (30). Taken together, our results show that although regulation can occur with a disrupted dimerization interface (M4) and in combination with loss of glycine binding to the second aptamer (M2+M3), proper tandem glycine riboswitch function and maximum gene expression appear to depend on the stabilization provided by interaptamer interactions and glycine binding to the first aptamer.

### The leader-linker kink-turn is required for tandem glycine riboswitch function.

To investigate the impact of the leader-linker interaction, the M5 mutation disrupts the kink-turn that forms the P0 helix found in over 90% of tandem glycine riboswitches (Fig. 1A) (26, 27). Previous inline probing, native gel analysis, small-angle X-ray scattering, and isothermal titration calorimetry experiments showed that this leader-linker interaction is formed independently of glycine and results in a more stable and compact structure that enhances glycine binding and interaptamer interactions (26–29). Truncations of the 5′ leader and mutations to the linker region that disrupt the formation of the P0 helix in the *V. cholerae* riboswitch significantly reduced the ligand-binding affinity of the RNA *in vitro*. The M5 mutation disrupting the kink-turn resulted in a complete loss of regulation. Combination of the M5 mutation with the glycine-binding mutation on the second aptamer (M2+M5) also resulted in loss of regulation and yielded activity similar to that of the M5 single mutant construct and the M1+M4 double mutant (Fig. 1B). This supports previous findings indicating that the leader-linker kink-turn plays a key role in riboswitch-mediated regulation and is important for glycine binding to the first aptamer.

### Control mutations to the glycine riboswitch behave as anticipated.

The M6 mutation destabilizes the base of the rho-independent terminator stem that forms when the riboswitch is in the "off" conformation in the absence of glycine. As expected, this mutation allowed constitutive expression of the *lacZ* reporter (Fig. 1). The slight (~2-fold) increase in β-galactosidase activity in the presence of glycine can be attributed to further destabilization of the terminator upon glycine binding and aptamer dimerization, as these domains remain intact.

We designed mutations M7 and M8 as controls that target regions on the first and second aptamers, respectively, in which RNA structure is conserved but nucleotide composition varies (Fig. 1) (11). Riboswitch regulation remained intact for both of these mutant constructs with activities comparable to that of the wild-type riboswitch under all of the glycine concentrations tested. The basal β-galactosidase activity of the M8 mutant construct in the absence of glycine was modestly higher than that of the wild-type and M7 mutant riboswitch constructs. Like the M2 single mutation, this mutation may also result in a slight change in the conformation of the second aptamer, possibly affecting its glycine-binding affinity, dimerization interactions, or the stability of the adjacent terminator stem when in the "off" conformation.

### *gcvT* expression of native locus recombinant strains reflects β-galactosidase assay data.

**FIG 2:**
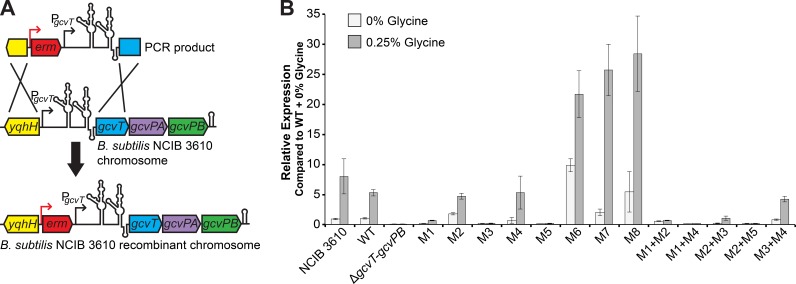
Construction and confirmation of recombinant glycine riboswitch *B. subtilis* strains. (A) Schematic of the strategy used to generate recombinant *B. subtilis* NCIB 3610 strains. The *gcvT* operon promoter (P_*gcvT*_), glycine riboswitch, and two ~500-bp regions flanking the promoter and riboswitch locus were PCR amplified from *B. subtilis* 168 genomic DNA. A PCR product in which an erythromycin resistance cassette (*erm*) was introduced into the intergenic region immediately upstream from the *gcvT* operon promoter was generated. Transformation of cloned PCR products into *B. subtilis* NCIB 3610 replaced the native copy of the glycine riboswitch with either a wild-type or a mutant recombinant version via double-crossover homologous recombination. (B) qRT-PCR quantification of the native *gcvT* transcript from each of the recombinant glycine riboswitch strains grown in M9 minimal medium with or without 0.25% glycine. For each strain/condition, *gcvT* expression was normalized to expression of the *nifU* control transcript. Graph depicts relative *gcvT* expression from each strain compared to *gcvT* expression from the wild-type (WT) recombinant grown in the absence of glycine (0% glycine). Error bars represent the standard error of the mean across three technical replicates.

In *B. subtilis*, the tandem glycine riboswitch turns on expression of the *gcvT* operon (encoding components of the glycine cleavage system, *gcvT*, *gcvPA*, and *gcvPB*, that catabolize glycine to ammonia, carbon dioxide, and one-carbon units utilized via the folate pool) in response to glycine (10, 11, 31). To explore the physiological role of the glycine riboswitch and determine whether *gcvT* operon expression changes resulting from mutations to the riboswitch impact *B. subtilis* fitness and growth, we replaced the native copy of the glycine riboswitch within the *B. subtilis* NCIB 3610 genome with either a wild-type or a mutant recombinant version (Fig. 2A). A recombinant strain in which the entire *gcvT-gcvPB* locus was deleted (Δ*gcvT*-*gcvPB*) was also generated to serve as a negative control. To confirm that our β-galactosidase assay results accurately represent changes in glycine riboswitch regulation and gene expression in the NCIB 3610 mutant recombinant strains, we performed quantitative reverse transcription (qRT)-PCR using primers within the *gcvT* coding region on log-phase total RNA from each of the recombinant strains grown in M9 minimal medium with or without 0.25% glycine (Fig. 2B).

These results are generally in good agreement with our reporter assay data (Fig. 1B) and further confirm the behavior of the *B. subtilis* tandem glycine riboswitch as an "on" switch that regulates *gcvT* operon expression via a transcription terminator in response to glycine. The mutations that retained riboswitch function in the β-galactosidase assays (M1, M2, M4, M2+M3) exhibited similar trends in regulatory activity and expression, as measured by qRT-PCR. Likewise, mutations M3, M5, M1+M4, and M2+M5 abrogated glycine riboswitch regulation and resulted in *gcvT* transcript expression levels comparable to that of the Δ*gcvT-gcvPB* operon deletion recombinant strain. The recombinant strain carrying the M6 mutation to the terminator exhibited elevated constitutive expression of the *gcvT* transcript regardless of the presence or absence of glycine. Also consistent with our previous data, the presence of the M1+M2 double mutation removed riboswitch regulation and resulted in basal *gcvT* transcript expression.

The only major discrepancy between the qRT-PCR results and our β-galactosidase assays is the behavior of the M3+M4 mutant. While the β-galactosidase assays suggest that the M3+M4 mutation resulted in loss of riboswitch function and slightly elevated basal expression, our qRT-PCR data indicate that riboswitch regulation is retained in the corresponding mutant recombinant strain. Additionally, the control recombinant strains containing mutations M7 and M8 exhibited *gcvT* transcript levels approximately 4- to 5-fold higher than that of the wild-type recombinant strain when grown in 0.25% glycine. This increase in expression is not represented in the reporter assay data, where the M7 and M8 mutant constructs yielded activities comparable to that of the wild-type construct in the presence of glycine. These discrepancies may be attributable to differences between *B. subtilis* strains 168 and NCIB 3610, to the greater sensitivity of qRT-PCR relative to β-galactosidase assays, or to the measurement of the native transcript rather than a translational reporter that does not include the entire transcript.

### *gcvT* operon expression affects glycine sensitivity and doubling time during planktonic growth.

Once the behavior of the glycine riboswitch mutations was confirmed in the recombinant *B. subtilis* NCIB 3610 strains, growth curve assays were performed with all of our recombinant strains in M9 minimal medium with increasing glycine concentrations (Table 1). High glycine concentrations are known to inhibit bacterial growth, as excess glycine interferes with cell wall biosynthesis (10, 32–36). In agreement with these observations, we observed a reduction in the maximum growth of all recombinant strains in the presence of glycine, and the doubling times of nearly all of the strains increased as the glycine concentration in the medium was increased (Table 1). A prolonged lag-phase and abnormal or little to no cell growth were also observed for most strains in 1% glycine; this is reflected in the high standard error reported for this glycine concentration (34).

**TABLE 1:** Doubling times of recombinant glycine riboswitch *B. subtilis* strains grown in increasing glycine concentrations^a^

Strain	Mean doubling time (min) ± SEM at glycine concn (%) of:			
	0	0.25	0.5	1
NCIB 3610 (parental)	70.0 ± 2.6	80.0 ± 2.7	82.8 ± 1.7	108.9 ± 5.8
WT	76.2 ± 2.3	87.9 ± 2.1	90.3 ± 3.9	110.6 ± 4.4
Δ*gcvT-gcvPB*	73.7 ± 2.7 (1.0)^b^	110.1 ± 4.8^c^ (1.3)	118.8 ± 5.1^c^ (1.3)	127.7 ± 9.8 (1.2)
M1	81.7 ± 5.2 (1.1)	98.7 ± 7.5 (1.1)	98.7 ± 11.0 (1.1)	116.5 ± 11.6 (1.1)
M2	69.3 ± 3.2 (0.9)	77.9 ± 4.5 (0.9)	84.1 ± 3.8 (0.9)	116.3 ± 12.5 (1.1)
M3	116.8 ± 7.8^c^ (1.5)	143.6 ± 4.0^c^ (1.6)	143.4 ± 0.0^c^ (1.6)	153.4 ± 5.0^c^ (1.4)
M4	76.7 ± 2.8 (1.0)	92.3 ± 2.5 (1.0)	94.3 ± 3.4 (1.0)	110.6 ± 5.0 (1.0)
M5	84.6 ± 2.8^c^ (1.1)	115.3 ± 7.8^c^ (1.3)	122.8 ± 7.0^c^ (1.4)	123.8 ± 13.8 (1.1)
M6	101.4 ± 7.4^c^ (1.3)	87.8 ± 1.7 (1.0)	96.1 ± 1.0 (1.1)	106.1 ± 7.7 (1.0)
M7	80.0 ± 5.7 (1.1)	91.9 ± 5.6 (1.1)	91.2 ± 6.3 (1.0)	106.7 ± 4.8 (1.0)
M8	78.2 ± 3.5 (1.0)	90.6 ± 3.5 (1.0)	97.3 ± 3.0 (1.1)	110.6 ± 10.3 (1.0)
M1+M2	80.0 ± 1.9 (1.1)	95.5 ± 4.5 (1.1)	103.1 ± 5.0 (1.1)	133.5 ± 6.8^c^ (1.2)
M1+M4	76.1 ± 6.1 (1.0)	107.2 ± 4.9^c^ (1.2)	106.7 ± 2.3^c^ (1.2)	126.9 ± 7.8 (1.2)
M2+M3	80.8 ± 2.8 (1.1)	89.6 ± 3.1 (1.0)	96.4 ± 3.8 (1.1)	116.1 ± 4.3 (1.1)
M2+M5	78.6 ± 1.8 (1.0)	100.4 ± 1.9^c^ (1.1)	112.3 ± 6.7^c^ (1.2)	126.8 ± 9.7 (1.2)
M3+M4	137.4 ± 6.5^c^ (1.8)	143.6 ± 4.0^c^ (1.6)	148.1 ± 2.4^c^ (1.6)	148.1 ± 2.4^c^ (1.3)

^a^ Strains were grown in M9 minimal medium with increasing glycine concentrations at 37°C with shaking (225 rpm) for 24 h. Each value is the mean of three or more independent experimental replicates ± the standard error of the mean across biological replicates.

^b^ In parentheses is the strain doubling time relative to that of the wild-type (WT) recombinant strain.

^c^ Strains grew significantly worse than the wild-type recombinant strain at the corresponding glycine concentration (*P* < 0.05).

Strikingly, the strain harboring the M6 mutation to the terminator stem grew approximately 1.3 times more slowly than the wild-type recombinant strain in medium lacking glycine. Furthermore, its doubling time decreased upon the addition of 0.25% glycine to the medium (101 to 88 min) and its growth was comparable to that of the wild-type recombinant strain under all glycine-supplemented conditions. As the M6 mutation results in constitutive expression of the *gcvT* operon, unnecessary expression of the glycine cleavage system in the absence of glycine appears to have adverse effects on cell growth, and this defect is rescued in the presence of glycine.

As expected, the Δ*gcvT-gcvPB* recombinant strain grew comparably to the wild-type recombinant in medium lacking glycine and demonstrated a significant increase in doubling time upon the addition of glycine to the medium, growing approximately 1.3 times more slowly than the wild-type recombinant in all of the glycine concentrations tested. This suggests that the *gcvT* glycine cleavage operon is important for glycine detoxification in *B. subtilis*. The M5, M1+M4, and M2+M5 mutant recombinant strains also displayed a modest increase in doubling time relative to the wild-type recombinant upon the addition of glycine to the medium. The increase in glycine sensitivity of these mutant recombinant strains during planktonic growth can be attributed to extremely low expression of the *gcvT* operon (Fig. 1B and 2B), further supporting the role that the *gcvT* operon plays in glycine detoxification.

No increase in glycine sensitivity was observed for the majority of the glycine riboswitch mutant recombinant strains that retained regulatory activity (M1, M2, M4, M7, M8, M2+M3). The doubling times of these strains remained consistent relative to that of the wild-type recombinant strain at all of the glycine concentrations tested (Table 1). The recombinant strain carrying the M1+M2 double mutation also grew comparably to the wild-type recombinant under all of the conditions tested. The basal expression from the M1+M2 construct appears to be sufficient for normal planktonic growth in high-glycine environments.

Interestingly, the strains carrying the M3 and M3+M4 mutations exhibited the longest doubling times, growing approximately 1.6 times more slowly than the wild-type recombinant strain in the presence and absence of glycine. This growth defect does not correlate well with gene expression, as measured by β-galactosidase activity and qRT-PCR. Each of the recombinant strains was created independently from the parental strain; thus, it is unlikely that this defect is due to some other common mutation. It is possible that components of the *gcvT* operon are important for metabolic processes not directly related to glycine and that the M3 and M3+M4 mutations further interfere with translation of the native operon transcript. Therefore, the glycine-independent planktonic growth defects observed for these strains may be due to disruptions to other fundamental biochemical processes important for growth.

While inhibition of cell growth was observed for all of the strains in increasing glycine concentrations as previously reported, only strains with mutations that completely abolish riboswitch function and result in extremely low *gcvT* operon expression appear to have greater glycine sensitivity than the wild-type recombinant strain during planktonic growth in minimal medium. The majority of these glycine-sensitive recombinant strains carry mutations that disrupt glycine binding to the first aptamer and/or first aptamer tertiary structure.

### Glycine cannot be utilized as a sole carbon source by *B. subtilis* NCIB 3610.

Others have demonstrated that the tandem glycine riboswitch-regulated operons *gcvT-gcvH* and *gcvP* facilitate the use of glycine as a carbon source in *Streptomyces griseus* (10). To examine whether *B. subtilis* could similarly metabolize glycine and assess whether this phenotype is sensitive to changes in *gcvT* operon expression resulting from glycine riboswitch mutations, we performed growth experiments with both solid and liquid minimal media in the presence and absence of glycine with limiting glucose. In contrast to *S. griseus*, *B. subtilis* NCIB 3610 is not able to grow on glycine as a sole carbon source (see Fig. S1 in the supplemental material). Of note, *gcvT-gcvH* and *gcvP* exist as separate operons and are under the control of separate tandem glycine riboswitches in *S. griseus*, whereas *gcvT* and *gcvPA-gcvPB* (two genes encoding separate subunits of the glycine decarboxylase GcvP) are colocated in the *B. subtilis* genome and are under the regulation of a single tandem glycine riboswitch. The predicted *B. subtilis* homolog of *gcvH* (also known as *yusH*) occurs elsewhere in the genome and does not appear to be glycine riboswitch controlled.

10.1128/mBio.01602-17.2 FIG S1  *B. subtilis* growth with glucose and glycine as carbon sources. (A) Growth curves of *B. subtilis* NCIB 3610 (parental strain) in M9 minimal medium with or without 0.25% glycine with various concentrations of glucose. Cultures were incubated at 37°C at 225 rpm for approximately 24 h, and OD_600_ measurements were recorded at time points indicated. (B) M9 minimal medium patch plates of selected *B. subtilis* NCIB 3610 strains with various carbon sources. Plates were incubated at 37°C for 1 week and photographed. Photographs taken after 48 h of incubation are shown; however, no significant changes in any of the plates were observed after 1 week. Download FIG S1, PDF file, 1 MB. Copyright © 2017 Babina et al. 2017 Babina et al. This content is distributed under the terms of the Creative Commons Attribution 4.0 International license.

### Glycine riboswitch mutants have reduced swarming motility in high-glycine environments.

To assess whether more complex *B. subtilis* phenotypes might be more sensitive readouts of glycine toxicity than planktonic growth doubling times, we next investigated the impact of riboswitch mutations and aberrant regulation of the glycine cleavage operon on swarming motility in the presence of glycine. Swarming motility is highly dependent on cellular differentiation into a swarming-proficient state and cellular contacts with surfaces and neighboring cells, all of which are at least partially mediated by peptidoglycan remodeling and cell wall synthesis and structural integrity (37, 38). Excess glycine can be misincorporated into bacterial cell walls, and high glycine concentrations inhibit the enzymes responsible for the addition of l-alanine into peptidoglycan precursors, resulting in weakened cell walls and premature lysis (33–36).

**FIG 3:**
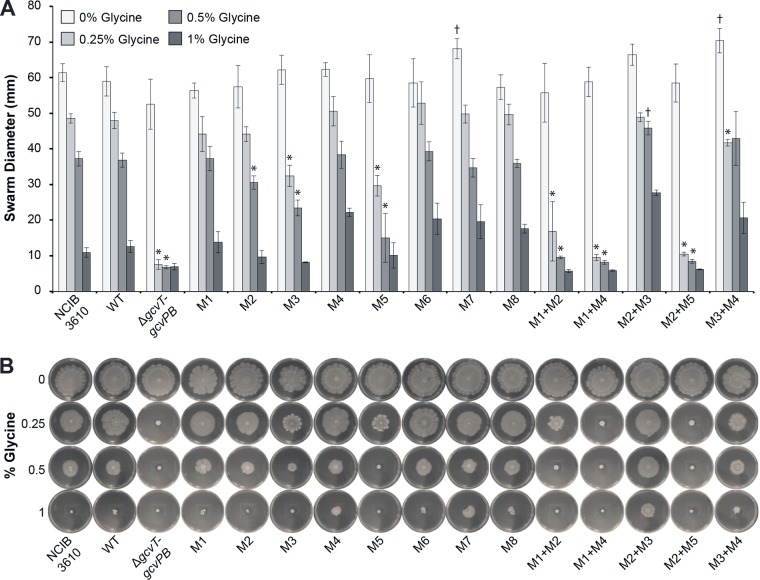
Swarming motility of recombinant glycine riboswitch *B. subtilis* strains in increasing glycine concentrations. M9 minimal medium swarm agar plates with various glycine concentrations were inoculated with each strain and incubated at 37°C for 48 h. (A) Swarm diameters of recombinant glycine riboswitch *B. subtilis* strains. Three measurements were taken for each plate and averaged. The values reported represent the mean of three independent experimental replicates; error bars represent the standard error of the mean. Asterisks indicate mutant recombinant strains that grew significantly worse than the wild-type (WT) recombinant in the corresponding glycine concentration. Daggers indicate mutant recombinant strains that grew significantly better than the wild-type recombinant in the corresponding glycine concentration (*P* < 0.05). Significance was not determined for measurements recorded at 1% glycine. (B) Representative photographs of swarming motility assays.

No significant difference in swarm diameter was observed for any of the strains in the absence of glycine. Similar to the cell growth rate, the swarm diameter of all of the strains decreased as the glycine concentration in the medium was increased (Fig. 3). Little to no migration was observed for all of the strains at 1% glycine. The large variation in swarm diameter seen at this concentration can be attributed to the prevalence of escape motile or flare mutants, which resulted in swarms with nonuniform diameters and is indicative of glycine toxicity. Consequently, significance was not determined for swarm diameter measurements at 1% glycine. Apart from differences in migration diameter, no other strain-specific or glycine concentration-dependent trends in swarm morphology were noted.

Strains with mutations that abolish riboswitch regulation and result in low downstream expression (M3, M5, M1+M2, M1+M4, M2+M5) demonstrated a significant reduction in swarming motility in the presence of glycine compared to the NCIB 3610 parental and wild-type recombinant strains (Fig. 3). The M1+M2, M1+M4, and M2+M5 double mutant recombinant strains exhibited the most severe glycine-sensitive swarm phenotypes that were comparable to those of the Δ*gcvT-gcvPB* recombinant strain. Strains that retain riboswitch function and/or have elevated constitutive operon expression (M1, M2, M4, M6, M7, M8, M2+M3, M3+M4) all behaved similarly to the wild-type recombinant and NCIB 3610 parental strains under all of the assay conditions tested.

It is unlikely that the defects observed during planktonic growth play a role in swarm migration distance. While the M3 mutant recombinant strain showed a significant swarm migration defect, the migration distance of the M3+M4 double mutant recombinant strain was comparable to that of the wild-type recombinant and the NCIB 3610 parental strains at all of the glycine concentrations tested. Similarly, while the doubling times of the M1+M2 mutant recombinant strain did not significantly differ from those of the wild-type recombinant strain during planktonic growth, the M1+M2 recombinant strain demonstrated significant glycine sensitivity during the swarming motility assays. Defects observed during planktonic cell growth can be distinct from those observed during static growth.

### Mutations to the glycine riboswitch inhibit biofilm formation in high-glycine environments.

To further assess the effects of disruptions to glycine riboswitch function and *gcvT* operon regulation on *B. subtilis* fitness in the presence of excess glycine, we analyzed mutant recombinant strain biofilm formation on solid medium, as well as the development of floating biofilms formed at the air-liquid interface (pellicle), as *B. subtilis* is a robust model organism for the study of biofilm development (for a review, see reference 39). A recent study demonstrated that interference with cell wall composition greatly disrupts *B. subtilis* biofilm formation (40) and excess glycine has been shown to inhibit *Streptococcus sobrinus* aggregation by way of reduced glucan-binding ability and weakened cell wall integrity (41).

**FIG 4:**
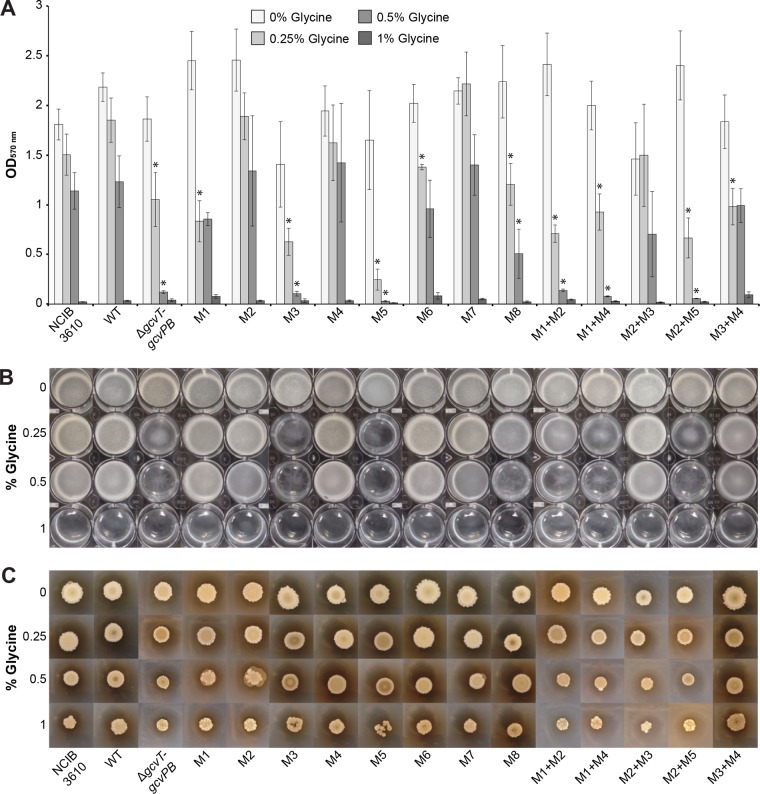
Biofilm formation of recombinant glycine riboswitch *B. subtilis* strains in increasing glycine concentrations. (A) Crystal violet staining of recombinant glycine riboswitch *B. subtilis* strain pellicle formation. Each value is the mean of six or more independent experimental replicates; error bars represent the standard error of the mean across biological replicates. Asterisks indicate mutant recombinant strains that grew significantly worse than the wild-type (WT) recombinant in the corresponding glycine concentration (*P* < 0.05). Significance was not determined for measurements recorded at 1% glycine. (B) Representative photographs of recombinant strain pellicle formation after 24 h of incubation at 37°C. (C) Representative photographs of recombinant strain colony biofilm formation on solid medium after 5 days of incubation at 30°C.

The extent of biofilm assembly on both solid and liquid media was reduced for all of the strains as the glycine concentration was increased. Little to no pellicle development was observed for all strains at 1% glycine; the pellicles that did form were very fragile and had a smooth surface (Fig. 4A and B). Consequently, significance is not reported for the crystal violet staining assays at this concentration. Similarly, biofilms that developed on solid medium with 1% glycine were small in diameter and lacked the wrinkled phenotype characteristic of robust *B. subtilis* biofilms (Fig. 4C).

The M3 and M5 single mutant recombinant strains, as well as the M1+M2, M1+M4, and M2+M5 double mutant recombinant strains, exhibited the greatest defects in biofilm formation in the presence of glycine on both solid and liquid media (Fig. 4). Pellicles were thinner and had significantly reduced or no surface wrinkling in comparison to the other strains. The solid-surface biofilms also displayed defects in colony wrinkling and were often dark brown in color in the presence of glycine, indicating sporulation or cell death due to harsh environmental conditions (42). The biofilms formed by these mutant recombinant strains were comparable to those of the Δ*gcvT-gcvPB* recombinant strain under all of the conditions tested.

The recombinant strains in which glycine riboswitch function remains intact and/or the *gcvT* operon is constitutively expressed (M1, M2, M4, M6, M7, M8, M2+M3, M3+M4) generated robust biofilms on both medium types, with morphologies similar to those produced by the wild-type recombinant and NCIB 3610 parental strains. It should be noted that while crystal violet staining is a simple and high-throughput method for quantifying pellicle biofilm formation, assay results are highly sensitive to minor changes in protocol or conditions and often yield a great range of variation between replicates (43, 44), as demonstrated by the high standard error reported. Nevertheless, these biofilm assay results and observed trends in glycine sensitivity are consistent with those from our swarming motility experiments.

## DISCUSSION

Since the initial discovery of the tandem glycine riboswitch, numerous studies have sought to elucidate the structure, dynamics, and selective advantages of the dual aptamer architecture. While informative, most of these investigations were limited to *in vitro* techniques and often yielded conflicting results. In this study, we examined the proposed models of tandem glycine riboswitch regulation in the context of its native locus within the *B. subtilis* genome and assessed how previously described interactions contribute to riboswitch function *in vivo* and overall cell fitness.

Mutations to the first aptamer (M1, M3, M5) resulted in the greatest reduction in downstream gene expression. All of these mutations directly or indirectly affect the structural stability and/or ligand-binding affinity of the first aptamer (11, 21, 23, 25–30). The M1 mutation to the glycine-binding domain on the first aptamer retained regulatory activity in response to glycine, although induced downstream expression was about one-tenth of that observed with the wild-type riboswitch. This suggests that glycine solely binding to the second aptamer can yield a regulatory response, but that glycine binding to the first aptamer is required for robust downstream gene expression. The first aptamer dimerization (M3) and leader-linker kink-turn (M5) mutations both completely abolished regulation and *gcvT* operon expression. This supports recent *in vitro* findings suggesting that aptamer dimerization is energetically linked to glycine binding in the first aptamer and that the ligand-binding affinity of the first aptamer is more sensitive to disruptions of the dimerization interface. These results also corroborate the importance of the P0/P1 helices for riboswitch dimerization and preorganization of the ligand-binding pockets (27, 30).

Mutations to the second aptamer had varying effects. Surprisingly, the M2 mutation to the glycine-binding domain on the second aptamer retained regulatory activity and expression levels comparable to those of the wild-type riboswitch. This reinforces the importance of glycine binding to the first aptamer for proper glycine riboswitch regulation and maximum downstream gene expression. Similarly, the M4 mutation to the dimerization domain on the second aptamer also retained regulatory function in response to glycine, although the resulting downstream gene expression was slightly less than that of the wild-type riboswitch. This finding supports previous studies suggesting that the second aptamer is capable of binding glycine despite disruptions to the dimerization interface (30). While differences in behavior between the M3 and M4 dimerization mutants may be due to the asymmetrical nature of the γ dimerization interface (different nucleotide mutations are required to disrupt the interaction for each aptamer), our other mutations corroborate a model where changes to the first aptamer have a bigger impact on gene expression.

Taken together, our results indicate that double ligand occupancy is not required and glycine can bind to either aptamer to elicit a regulatory response *in vivo*. However, glycine binding to the first aptamer in combination with the leader-linker kink-turn and interaptamer interactions is necessary for optimal tandem glycine riboswitch-mediated regulation, maximum *gcvT* operon expression, and subsequent *B. subtilis* survival in high-glycine environments. These findings offer new physiological insights into the decade-old dual aptamer debate and reinforce the hypothesis that the tandem architecture has been conserved against evolution not necessarily for enhanced ligand specificity and/or more digital gene control but because the complex tertiary structure mediated by the presence of and interactions between the two aptamers is important for promoting glycine binding and conformational changes of the expression platform (12, 27, 30). The recently proposed models of singlet glycine riboswitch regulation further support this theory. These single aptamer riboswitches bind glycine with affinities comparable to those with the dual aptamer structure; however, they are not true structural “singlets.” Interactions with stem-loop “ghost aptamers” are required for singlet riboswitch structural stability and regulation, although these interactions may be less complex and/or distinct from those found with the tandem aptamer architecture (12).

While we demonstrate that glycine binding to the second aptamer is not necessary to elicit the maximum regulatory response *in vivo*, previous bioinformatic analyses indicate that the glycine-binding domains of both aptamers are equally well conserved (45). This suggests that maintaining two functional ligand-binding domains may confer some evolutionary advantage over the singlet aptamer conformation. Despite the fact that our study does not directly suggest a cooperative mechanism of ligand binding, as this behavior is difficult to discern *in vivo*, the possibility that the tandem glycine riboswitch acts cooperatively within the cell cannot be precluded.

Although our overall conclusions are in agreement with the current model of tandem glycine riboswitch function, some of our data do not directly align with those from previous reports. Namely, we find that disruptions to the glycine-binding pocket on the second aptamer (M2) do not compromise riboswitch function or downstream expression *in vivo*, whereas *in vitro* studies with the *V. cholerae* riboswitch show that homologous mutations to the second aptamer have a more severe impact on tandem riboswitch ligand-binding affinity (30). A similar trend is observed with our dimerization mutations. Our data suggest that mutations to the γ dimerization interface on the first aptamer (M3) completely abrogate riboswitch function, while mutations to the dimerization interface on the second aptamer (M4) still retain regulatory activity, albeit with reduced downstream expression. Past work with homologous *V. cholerae* riboswitch mutant constructs demonstrate that disrupting the γ dimerization domain on the second aptamer results in the greatest reduction in dimerization affinity between the two aptamers (30).

The discrepancies between our results and those obtained with the *V. cholerae* riboswitch are most likely due to differences between *in vitro* and *in vivo* experimental conditions. Our experiments utilized the full-length *B. subtilis* tandem glycine riboswitch, and our conclusions are based on *in vivo* gene expression and reporter enzyme activity levels, whereas previous investigations directly measured glycine binding and dimerization affinities *in vitro* using both *cis* (full-length) and *trans V. cholerae* tandem glycine riboswitch constructs (30). Additionally, though the tandem glycine riboswitches found in *B. subtilis* and *V. cholerae* are homologous, they are not identical and thus may not behave in the same manner in both *in vivo* and *in vitro* environments. For example, the *B. subtilis* tandem glycine riboswitch is followed by a rho-independent terminator and its regulatory mechanism of action is well characterized; the expression platform and mechanism by which the *V. cholerae* tandem glycine riboswitch regulates gene expression *in vivo* has not been characterized in detail (11). Consequently, while general conclusions can be drawn across studies, direct comparisons may not be applicable.

This work offers novel insights into tandem glycine riboswitch behavior within the context of its native locus and highlights the advantages of combining *in vivo* and *in vitro* techniques to obtain a more comprehensive understanding of riboswitch function from biochemical, biophysical, physiological, and evolutionary perspectives. Using *B. subtilis* as a model organism, we demonstrate the importance of proper tandem glycine riboswitch function as a genetic "on" switch for *gcvT* operon expression and optimal cell growth in both the presence and absence of glycine. The effects subtle point mutations to the tandem glycine riboswitch have on regulation, gene expression, and communal bacterial behaviors such as swarming and biofilm formation reinforce the potential of riboswitches as antimicrobial drug targets. Knowledge of how cells respond to the loss of riboswitch regulation allows for a more informed and directed approach to the design of riboswitch-targeting antibiotics and provides insight into how resistance to such compounds may evolve in the future.

## MATERIALS AND METHODS

### β-Galactosidase activity assay plasmid and strain construction.

*B. subtilis* integration vector pDG1728 was modified to allow for a translational fusion between the glycine riboswitch constructs and *lacZ* (46). Briefly, the region containing the *spoVG* ribosome-binding site and the *lacZ* start codon was removed using the EcoRI and BamHI restriction sites and replaced with a cassette that allowed for a translational fusion with the *lacZ* reporter when cloning with BamHI (5′ GAATTCTACGACAAATTGCAAAAATAATGTTGTCCTTTTAAATAAGATCTGATAAAATGTGAACTAAGCTTCTAGGATCC 3′ [underlining indicates EcoRI and BamHI restriction sites, respectively]).

To generate the glycine riboswitch-*lacZ* translational fusion constructs, the region containing the *gcvT* operon promoter, wild-type glycine riboswitch, and ribosome-binding site and first nine codons of *gcvT* was PCR amplified from *B. subtilis* 168 genomic DNA (GenBank accession number AL009126; complement of 2549307 to 2549704) (47) with primers containing EcoRI and BamHI restriction sites (Table S1). After digestion, the PCR product was cloned into the modified pDG1728 vector digested with the same enzymes. Mutations to the glycine riboswitch were obtained by site-directed mutagenesis or PCR assembly (Table S1). All plasmids were verified via Sanger sequencing.

10.1128/mBio.01602-17.3 TABLE S1  Oligonucleotides used in this study. For each primer pair, the forward primer is listed first and the reverse primer is listed second. Download TABLE S1, PDF file, 0.1 MB. Copyright © 2017 Babina et al. 2017 Babina et al. This content is distributed under the terms of the Creative Commons Attribution 4.0 International license.

Reporter constructs were transformed into *B. subtilis* 168 as described previously (48, 49). Transformants were screened for resistance to spectinomycin (100 μg/ml), sensitivity to erythromycin (0.5 μg/ml), and loss of amylase activity (plating on tryptose blood agar base plus 1% starch and staining with Gram’s iodine solution; Sigma-Aldrich) to ensure proper integration of the *lacZ* reporter constructs into the *amyE* locus.

### β-Galactosidase activity assays.

*B. subtilis* 168 *lacZ* reporter strains were grown from single colonies in 2 ml of M9 minimal medium containing 1% glucose, 50 μg/ml tryptophan, and 100 μg/ml spectinomycin for approximately 24 h at 37°C with shaking (225 rpm). These cultures were diluted 1:10 into 2 ml of M9 minimal medium containing 1% glucose, 50 μg/ml tryptophan, and 100 μg/ml spectinomycin with various glycine concentrations (0, 0.25, 0.5, and 1% [wt/vol] or approximately 0, 33.3, 66.6, and 133.2 mM) and grown for approximately 10 h at 37°C with shaking (225 rpm). Cells (1.5 ml) were harvested and resuspended in 1 ml of Z buffer (50 mM Na_2_HPO_4_, 40 mM NaH_2_PO_4_, 10 mM KCl, 1 mM MgSO_4_, 50 mM 2-mercaptoethanol) plus 100 μg/ml spectinomycin. The optical density at 600 nm (OD_600_) was measured as a 1:10 dilution of a cell suspension in Z buffer. Samples with a final OD_600_ of <0.5 were discarded. β-Galactosidase activity assays were performed as previously described, using 0.5 ml of cell suspensions, and activities in Miller units were calculated as follows (50): Activity (Miller units) = 1,000 × {*A*_420_/[Δ*t* (minutes) × *A*_600_ × Volume (milliliters)]}. The values reported represent three or more independent replicates; error bars represent the standard error of the mean across biological replicates.

### Recombinant glycine riboswitch strain construction.

To generate the recombinant strains for the growth curve, swarming motility, and biofilm assays, the *gcvT* operon promoter, wild-type glycine riboswitch, and two ~500-bp regions of homology flanking either side of the promoter and riboswitch region were PCR amplified from *B. subtilis* 168 genomic DNA (GenBank accession number AL009126; complement of 2549705 to 2550291 for the 5′ flanking ~500-bp region of homology, complement of 2549075 to 2549704 for the region containing the promoter, riboswitch, and 3′ flanking ~500-bp region of homology). An erythromycin resistance cassette was PCR amplified from *B. subtilis* integration vector pDG1663 (GenBank accession number U46200; 3930 to 5160) (46). PCR assembly with Phusion High-Fidelity DNA polymerase (Thermo, Fisher Scientific) was then used to generate recombinant PCR products in which the erythromycin resistance cassette was inserted immediately upstream from the *gcvT* operon promoter, between the two regions of homology amplified from *B. subtilis* genomic DNA (Table S1). To generate the Δ*gcvT-gcvPB* construct, ~500 bp immediately downstream from the *gcvPB* coding region was PCR amplified from *B. subtilis* 168 genomic DNA for the 3′ region of homology (GenBank accession number AL009126; complement of 2544924 to 2545409). A double rho-independent terminator construct was PCR amplified from pYH213 (51) (same sequence as GenBank accession number AY599227; 631 to 768) (52) and appended onto the 3′ end of the erythromycin resistance cassette to prevent any readthrough from the resistance cassette promoter. The complete Δ*gcvT-gcvPB* recombinant construct was then assembled as described above. All assembly PCR constructs were polyadenylated with *Taq* DNA polymerase (NEB), gel purified, and cloned into the pCR2.1 or pCR4 TOPO-TA vector (Invitrogen). Mutations to the glycine riboswitch were obtained by site-directed mutagenesis or PCR assembly (Table S1). All plasmids were verified via Sanger sequencing.

Recombinant pCR2.1 or pCR4 constructs were transformed into *B. subtilis* NCIB 3610 as described previously (48, 49). Transformants were screened for resistance to erythromycin (0.5 μg/ml). Integration of the complete recombinant construct within the *gcvT* locus and the presence of the riboswitch mutations of interest were verified via PCR and Sanger sequencing.

### Quantitative RT-PCR.

Total RNA was extracted from early- to mid-log-phase (OD_600_ of ~0.4 to 0.6) *B. subtilis* NCIB 3610 strains grown in M9 minimal medium containing 1% glucose with or without 0.25% glycine (plus 0.5 μg/ml erythromycin for recombinant strains) at 37°C with shaking (225 rpm). To remove genomic DNA, 5 μg of total RNA was treated with RQ1 DNase (Promega) at 37°C for 40 min; this was followed by incubation at 98°C for 2 min to heat inactivate the enzyme, phenol-chloroform extraction, and ethanol precipitation. RT was performed with random hexamers and SuperScript III (Invitrogen) in accordance with the manufacturer’s protocol, and the resulting cDNA served as the template for quantitative PCR with an ABI 7500 Fast real-time PCR system (Thermo, Fisher Scientific) and SYBR green detection. Glycine cleavage operon transcript levels were quantified using primers targeting the *gcvT* coding region, and quantification of *nifU* was used as a normalization control (Table S1) (53). To confirm effective removal of contaminating DNA, experiments were also conducted with reactions lacking reverse transcriptase. Error bars represent the standard error of the mean across three technical replicates propagated using standard calculations (54).

### Growth curves.

*B. subtilis* NCIB 3610 strains were grown from single colonies in 2 ml of M9 minimal medium containing 1% glucose (plus 0.5 μg/ml erythromycin for recombinant strains) for approximately 16 h at 37°C with shaking (225 rpm). These starter cultures were used to inoculate 0.5 ml of M9 minimal medium containing 1% glucose (plus 0.5 μg/ml erythromycin for recombinant strains) cultures with various glycine concentrations (0, 0.25, 0.5, and 1% [wt/vol] or approximately 0, 33.3, 66.6, and 133.2 mM) in sterile nontreated 24-well cell culture plates to a starting OD_600_ of approximately 0.2. Plates were incubated at 37°C with shaking (225 rpm) for 24 h. OD_600_s were recorded at 1-h intervals with a SpectraMax M3 Multi-Mode Microplate Reader (Molecular Devices). Doubling times were calculated as previously described, by taking the inverse of the slope of ln(OD_600_) in exponential-phase readings (55). The values reported represent three or more independent replicates; error reported represents the standard error of the mean across biological replicates. To determine the significance, mutant recombinant strain doubling times were compared to those of the wild-type recombinant strain at each glycine concentration using Welch’s single-tailed *t* test in Microsoft Excel. Values were considered significantly different if the *P* value was <0.05. For details of the growth curve assays performed with various carbon sources, see Text S1.

10.1128/mBio.01602-17.1 TEXT S1  Supplemental materials and methods used in this study. Download TEXT S1, PDF file, 0.1 MB. Copyright © 2017 Babina et al. 2017 Babina et al. This content is distributed under the terms of the Creative Commons Attribution 4.0 International license.

### Swarming motility assays.

*B. subtilis* NCIB 3610 starter cultures were prepared as described above and grown to similar stationary-phase OD_600_s, and 5 μl of each culture was spotted onto the center of swarming motility plates of M9 minimal medium containing 1% glucose plus 0.7% agar with the above glycine concentrations (37, 38). Plates were incubated at 37°C for 48 h and then photographed with a Samsung WB380F digital camera. The diameter of swarm motility growth was measured and recorded for each sample using FIJI software (56); three measurements of each plate were taken and averaged. The values reported represent three independent replicates; error bars represent the standard error of the mean across biological replicates. To determine the significance, mutant recombinant strain swarm diameters were compared to those of the wild-type recombinant strain at each glycine concentration using Welch’s single-tailed *t* test in Microsoft Excel. Values were considered significantly different if the *P* value was <0.05. Representative photographs are shown.

### Crystal violet staining.

*B. subtilis* NCIB 3610 starter cultures were prepared as described above and grown to similar stationary-phase OD_600_ values, and 1 μl of each starter culture was used to inoculate 100 μl of MSgg minimal medium (5 mM potassium phosphate [pH 7], 100 mM morpholinepropanesulfonic acid [MOPS; pH 7], 2 mM MgCl_2_, 700 μM CaCl_2_, 50 μM MnCl_2_, 50 μM FeCl_3_, 1 μM ZnCl_2_, 2 μM thiamine, 0.5% glycerol, 0.5% glutamate, 50 μg/ml tryptophan, and 50 μg/ml phenylalanine plus 0.5 μg/ml erythromycin for recombinant strains) cultures with the above glycine concentrations in sterile nontreated 96-well cell culture plates (57). Plates were incubated at 37°C without agitation for 30 h. Following incubation, culture supernatant was removed and discarded and the wells were washed twice with 100 μl of 1× phosphate-buffered saline (137 mM NaCl, 2.7 mM KCl, 10 mM Na_2_HPO_4_, 1.8 mM KH_2_PO_4_, pH 7.4). Plates were air dried for 20 min, and the remaining surface-attached cells were stained with 100 μl of 0.1% Gram’s crystal violet (BD Biosciences) for 20 min. The stain was then removed, and plates were washed three times with 100 μl of sterile water and allowed to air dry for at least 30 min. Biofilm-associated crystal violet was then resolubilized in 100 μl of 96% ethanol and incubated at room temperature for 10 min. For quantification, resolubilized samples were diluted 1:10 into 96% ethanol and OD_570_ measurements were made with a SpectraMax M3 Multi-Mode Microplate Reader (Molecular Devices). Wells incubated with cell-free medium were washed and stained as described above to serve as a negative control and blank; the OD_570_ values of the cell-free wells were averaged and subtracted from the OD_570_ values of each sample (modified from reference 58). The values reported represent six or more independent replicates; error bars represent the standard error of the mean across biological replicates. To determine the significance, mutant recombinant strain crystal violet OD_570_ values were compared to those of the wild-type recombinant strain at each glycine concentration using Welch’s single-tailed *t* test in Microsoft Excel. Values were considered significantly different if the *P* value was <0.05.

### Pellicle assays.

*B. subtilis* NCIB 3610 starter cultures were prepared as described above and grown to similar stationary-phase OD_600_ values, and 1 μl of each culture was used to inoculate 0.5 ml MSgg minimal medium cultures (plus 0.5 μg/ml erythromycin for recombinant strains) with the above glycine concentrations in sterile nontreated 24-well cell culture plates (57). Plates were incubated at 37°C without agitation for 40 h. Wells were photographed with a Samsung WB380F digital camera at 24, 26, 28, 30, and 40 h post-inoculation. This assay was repeated three independent times for each strain; representative photographs at 24 h post-inoculation are shown.

### Colony biofilm assays.

*B. subtilis* NCIB 3610 starter cultures were prepared as described above and grown to similar stationary-phase OD_600_ values, and 10 μl of each culture was spotted onto the center of plates of MSgg minimal medium containing 1.5% agar with the above glycine concentrations (57). Plates were incubated at 30°C for 5 days and then photographed with a Samsung WB380F digital camera. This assay was repeated two independent times for each strain; representative photographs are shown.

## References

[1] Winkler WC, Breaker RR 2005 Regulation of bacterial gene expression by riboswitches. Annu Rev Microbiol 59:487–517. doi:10.1146/annurev.micro.59.030804.121336. 16153177

[2] Serganov A, Nudler E 2013 A decade of riboswitches. Cell 152:17–24. doi:10.1016/j.cell.2012.12.024. 23332744 PMC4215550

[3] Breaker RR 2012 Riboswitches and the RNA world. Cold Spring Harb Perspect Biol 4:a003566. doi:10.1101/cshperspect.a003566. 21106649 PMC3281570

[4] Blount KF, Wang JX, Lim J, Sudarsan N, Breaker RR 2007 Antibacterial lysine analogs that target lysine riboswitches. Nat Chem Biol 3:44–49. doi:10.1038/nchembio842. 17143270

[5] Deigan KE, Ferré-D’Amaré AR 2011 Riboswitches: discovery of drugs that target bacterial gene-regulatory RNAs. Acc Chem Res 44:1329–1338. doi:10.1021/ar200039b. 21615107 PMC3193592

[6] Mulhbacher J, St-Pierre P, Lafontaine DA 2010 Therapeutic applications of ribozymes and riboswitches. Curr Opin Pharmacol 10:551–556. doi:10.1016/j.coph.2010.07.002. 20685165

[7] Lünse CE, Schüller A, Mayer G 2014 The promise of riboswitches as potential antibacterial drug targets. Int J Med Microbiol 304:79–92. doi:10.1016/j.ijmm.2013.09.002. 24140145

[8] Ster C, Allard M, Boulanger S, Boulet ML, Mulhbacher J, Lafontaine DA, Marsault E, Lacasse P, Malouin F 2013 Experimental treatment of *Staphylococcus aureus* bovine intramammary infection using a guanine riboswitch ligand analog. J Dairy Sci 96:1000–1008. doi:10.3168/jds.2012-5890. 23245959

[9] Mulhbacher J, Brouillette E, Allard M, Fortier LC, Malouin F, Lafontaine DA 2010 Novel riboswitch ligand analogs as selective inhibitors of guanine-related metabolic pathways. PLoS Pathog 6:e1000865. doi:10.1371/journal.ppat.1000865. 20421948 PMC2858708

[10] Tezuka T, Ohnishi Y 2014 Two glycine riboswitches activate the glycine cleavage system essential for glycine detoxification in *Streptomyces griseus*. J Bacteriol 196:1369–1376. doi:10.1128/JB.01480-13. 24443533 PMC3993345

[11] Mandal M, Lee M, Barrick JE, Weinberg Z, Emilsson GM, Ruzzo WL, Breaker RR 2004 A glycine-dependent riboswitch that uses cooperative binding to control gene expression. Science 306:275–279. doi:10.1126/science.1100829. 15472076

[12] Ruff KM, Muhammad A, McCown PJ, Breaker RR, Strobel SA 2016 Singlet glycine riboswitches bind ligand as well as tandem riboswitches. RNA 22:1728–1738. doi:10.1261/rna.057935.116. 27659053 PMC5066625

[13] Ames TD, Breaker RR 2011 Bacterial aptamers that selectively bind glutamine. RNA Biol 8:82–89. doi:10.4161/rna.8.1.13864. 21282981 PMC3127080

[14] Welz R, Breaker RR 2007 Ligand binding and gene control characteristics of tandem riboswitches in *Bacillus anthracis*. RNA 13:573–582. doi:10.1261/rna.407707. 17307816 PMC1831863

[15] Poiata E, Meyer MM, Ames TD, Breaker RR 2009 A variant riboswitch aptamer class for S-adenosylmethionine common in marine bacteria. RNA 15:2046–2056. doi:10.1261/rna.1824209. 19776155 PMC2764483

[16] Zhou H, Zheng C, Su J, Chen B, Fu Y, Xie Y, Tang Q, Chou SH, He J 2016 Characterization of a natural triple-tandem c-di-GMP riboswitch and application of the riboswitch-based dual-fluorescence reporter. Sci Rep 6:20871. doi:10.1038/srep20871. 26892868 PMC4759541

[17] Trausch JJ, Ceres P, Reyes FE, Batey RT 2011 The structure of a tetrahydrofolate-sensing riboswitch reveals two ligand binding sites in a single aptamer. Structure 19:1413–1423. doi:10.1016/j.str.2011.06.019. 21906956 PMC3196276

[18] Gao A, Serganov A 2014 Structural insights into recognition of c-di-AMP by the *ydaO* riboswitch. Nat Chem Biol 10:787–792. doi:10.1038/nchembio.1607. 25086507 PMC4294798

[19] Ren A, Patel DJ 2014 c-di-AMP binds the *ydaO* riboswitch in two pseudo-symmetry–related pockets. Nat Chem Biol 10:780–786. doi:10.1038/nchembio.1606. 25086509 PMC4217635

[20] Lipfert J, Das R, Chu VB, Kudaravalli M, Boyd N, Herschlag D, Doniach S 2007 Structural transitions and thermodynamics of a glycine-dependent riboswitch from *Vibrio cholerae*. J Mol Biol 365:1393–1406. doi:10.1016/j.jmb.2006.10.022. 17118400 PMC1941672

[21] Kwon M, Strobel SA 2008 Chemical basis of glycine riboswitch cooperativity. RNA 14:25–34. doi:10.1261/rna.771608. 18042658 PMC2151043

[22] Lipfert J, Sim AYL, Herschlag D, Doniach S 2010 Dissecting electrostatic screening, specific ion binding, and ligand binding in an energetic model for glycine riboswitch folding. RNA 16:708–719. doi:10.1261/rna.1985110. 20194520 PMC2844619

[23] Erion TV, Strobel SA 2011 Identification of a tertiary interaction important for cooperative ligand binding by the glycine riboswitch. RNA 17:74–84. doi:10.1261/rna.2271511. 21098652 PMC3004068

[24] Huang L, Serganov A, Patel DJ 2010 Structural insights into ligand recognition by a sensing domain of the cooperative glycine riboswitch. Mol Cell 40:774–786. doi:10.1016/j.molcel.2010.11.026. 21145485 PMC3726718

[25] Butler EB, Xiong Y, Wang J, Strobel SA 2011 Structural basis of cooperative ligand binding by the glycine riboswitch. Chem Biol 18:293–298. doi:10.1016/j.chembiol.2011.01.013. 21439473 PMC3076126

[26] Kladwang W, Chou FC, Das R 2012 Automated RNA structure prediction uncovers a kink-turn linker in double glycine riboswitches. J Am Chem Soc 134:1404–1407. doi:10.1021/ja2093508. 22192063

[27] Sherman EM, Esquiaqui J, Elsayed G, Ye JD 2012 An energetically beneficial leader-linker interaction abolishes ligand-binding cooperativity in glycine riboswitches. RNA 18:496–507. doi:10.1261/rna.031286.111. 22279151 PMC3285937

[28] Baird NJ, Ferré-D’Amaré AR 2013 Modulation of quaternary structure and enhancement of ligand binding by the K-turn of tandem glycine riboswitches. RNA 19:167–176. doi:10.1261/rna.036269.112. 23249744 PMC3543082

[29] Esquiaqui JM, Sherman EM, Ionescu SA, Ye JD, Fanucci GE 2014 Characterizing the dynamics of the leader–linker interaction in the glycine riboswitch with site-directed spin labeling. Biochemistry 53:3526–3528. doi:10.1021/bi500404b. 24849816 PMC4059530

[30] Ruff KM, Strobel SA 2014 Ligand binding by the tandem glycine riboswitch depends on aptamer dimerization but not double ligand occupancy. RNA 20:1775–1788. doi:10.1261/rna.047266.114. 25246650 PMC4201829

[31] Kikuchi G, Motokawa Y, Yoshida T, Hiraga K 2008 Glycine cleavage system: reaction mechanism, physiological significance, and hyperglycinemia. Proc Jpn Acad Ser B Phys Biol Sci 84:246–263. doi:10.2183/pjab.84.246. PMC3666648 18941301

[32] Snell EE, Guirard BM 1943 Some interrelationships of pyridoxine, alanine and glycine in their effect on certain lactic acid bacteria. Proc Natl Acad Sci U S A 29:66–73. doi:10.1073/pnas.29.2.66. 16588604 PMC1078561

[33] Gordon J, Hall RA, Stickland LH 1951 The kinetics of the lysis of *Bacterium coli* by glycine. J Hyg (Lond) 49:169–174. doi:10.1017/S0022172400044065. 14880688 PMC2235021

[34] Hishinuma F, Izaki K, Takahashi H 1969 Effects of glycine and d-amino acids on growth of various microorganisms. Agric Biol Chem 33:1577–1586. doi:10.1271/bbb1961.33.1577.

[35] Hishinuma F, Izaki K, Takahashi H 1971 Inhibition of l-alanine adding enzyme by glycine. Agric Biol Chem 35:2050–2058.

[36] Hammes W, Schleifer KH, Kandler O 1973 Mode of action of glycine on the biosynthesis of peptidoglycan. J Bacteriol 116:1029–1053. 4200845 10.1128/jb.116.2.1029-1053.1973 PMC285483

[37] Kearns DB, Losick R 2003 Swarming motility in undomesticated *Bacillus subtilis*. Mol Microbiol 49:581–590. doi:10.1046/j.1365-2958.2003.03584.x. 12864845

[38] Copeland MF, Weibel DB 2009 Bacterial swarming: a model system for studying dynamic self-assembly. Soft Matter 5:1174–1187. doi:10.1039/B812146J. 23926448 PMC3733279

[39] Cairns LS, Hobley L, Stanley-Wall NR 2014 Biofilm formation by *Bacillus subtilis*: new insights into regulatory strategies and assembly mechanisms. Mol Microbiol 93:587–598. doi:10.1111/mmi.12697. 24988880 PMC4238804

[40] Bucher T, Oppenheimer-Shaanan Y, Savidor A, Bloom-Ackermann Z, Kolodkin-Gal I 2015 Disturbance of the bacterial cell wall specifically interferes with biofilm formation. Environ Microbiol Rep 7:990–1004. doi:10.1111/1758-2229.12346. 26472159

[41] Luengpailin J, Doyle RJ 2000 Glycine prevents the phenotypic expression of streptococcal glucan-binding lectin. Biochim Biophys Acta 1474:212–218. doi:10.1016/S0304-4165(00)00010-6. 10742601

[42] Sandman K, Kroos L, Cutting S, Youngman P, Losick R 1988 Identification of the promoter for a spore coat protein gene in *Bacillus subtilis* and studies on the regulation of its induction at a late stage of sporulation. J Mol Biol 200:461–473. doi:10.1016/0022-2836(88)90536-0. 3135411

[43] Kwasny SM, Opperman TJ 2010 Static biofilm cultures of Gram-positive pathogens grown in a microtiter format used for anti-biofilm drug discovery. Curr Protoc Pharmacol Chapter 13A:Unit 13A.8. 10.1002/0471141755.ph13a08s50 PMC3272335 22294365

[44] Li X, Yan Z, Xu J 2003 Quantitative variation of biofilms among strains in natural populations of *Candida albicans*. Microbiology 149:353–362. doi:10.1099/mic.0.25932-0. 12624197

[45] Barrick JE, Breaker RR 2007 The distributions, mechanisms, and structures of metabolite-binding riboswitches. Genome Biol 8:R239. doi:10.1186/gb-2007-8-11-r239. 17997835 PMC2258182

[46] Guérout-Fleury AM, Frandsen N, Stragier P 1996 Plasmids for ectopic integration in *Bacillus subtilis*. Gene 180:57–61. doi:10.1016/S0378-1119(96)00404-0. 8973347

[47] Kunst F, Ogasawara N, Moszer I, Albertini AM, Alloni G, Azevedo V, Bertero MG, Bessières P, Bolotin A, Borchert S, Borriss R, Boursier L, Brans A, Braun M, Brignell SC, Bron S, Brouillet S, Bruschi CV, Caldwell B, Capuano V, Carter NM, Choi SK, Cordani JJ, Connerton IF, Cummings NJ, Daniel RA, Denziot F, Devine KM, Düsterhöft A, Ehrlich SD, Emmerson PT, Entian KD, Errington J, Fabret C, Ferrari E, Foulger D, Fritz C, Fujita M, Fujita Y, Fuma S, Galizzi A, Galleron N, Ghim SY, Glaser P, Goffeau A, Golightly EJ, Grandi G, Guiseppi G, Guy BJ, Haga K, et al. 1997 The complete genome sequence of the Gram-positive bacterium *Bacillus subtilis*. Nature 390:249–256. doi:10.1038/36786. 9384377

[48] Yasbin RE, Wilson GA, Young FE 1975 Transformation and transfection in lysogenic strains of *Bacillus subtilis*: evidence for selective induction of prophage in competent cells. J Bacteriol 121:296–304. 803952 10.1128/jb.121.1.296-304.1975 PMC285643

[49] Jarmer H, Berka R, Knudsen S, Saxild HH 2002 Transcriptome analysis documents induced competence of *Bacillus subtilis* during nitrogen limiting conditions. FEMS Microbiol Lett 206:197–200. doi:10.1111/j.1574-6968.2002.tb11009.x. 11814663

[50] Miller J 1992 A short course in bacterial genetics. Cold Spring Harbor Laboratory Press.

[51] Yakhnin H, Yakhnin AV, Babitzke P 2015 Ribosomal protein L10(L12)4 autoregulates expression of the *Bacillus subtilis rplJL* operon by a transcription attenuation mechanism. Nucleic Acids Res 43:7032–7043. doi:10.1093/nar/gkv628. 26101249 PMC4538822

[52] Choi K-H, Gaynor JB, White KG, Lopez C, Bosio CM, Karkhoff-Schweizer RR, Schweizer HP 2005 A Tn7-based broad-range bacterial cloning and expression system. Nat Methods 2:443–448. doi:10.1038/nmeth765. 15908923

[53] Reiter L, Kolstø AB, Piehler AP 2011 Reference genes for quantitative, reverse transcription PCR in *Bacillus cereus* group strains throughout the bacterial life cycle. J Microbiol Methods 86:210–217. doi:10.1016/j.mimet.2011.05.006. 21620905

[54] Taylor JR 1997 An introduction to error analysis: the study of uncertainties in physical measurements. University Science Books, Sausalito, CA.

[55] Rubinow SI 1975 Introduction to mathematical biology. John Wiley & Sons, New York, NY.

[56] Schindelin J, Arganda-Carreras I, Frise E, Kaynig V, Longair M, Pietzsch T, Preibisch S, Rueden C, Saalfeld S, Schmid B, Tinevez JY, White DJ, Hartenstein V, Eliceiri K, Tomancak P, Cardona A 2012 Fiji: an open-source platform for biological-image analysis. Nature Methods 9:676–682. doi:10.1038/nmeth.2019. 22743772 PMC3855844

[57] Branda SS, González-Pastor JE, Ben-Yehuda S, Losick R, Kolter R 2001 Fruiting body formation by *Bacillus subtilis*. Proc Natl Acad Sci U S A 98:11621–11626. doi:10.1073/pnas.191384198. 11572999 PMC58779

[58] Kayumov AR, Khakimullina EN, Sharafutdinov IS, Trizna EY, Latypova LZ, Lien HT, Margulis AB, Bogachev MI, Kurbangalieva AR 2015 Inhibition of biofilm formation in *Bacillus subtilis* by new halogenated furanones. J Antibiot 68:297–301. doi:10.1038/ja.2014.143. 25335695

[59] Irnov I, Sharma CM, Vogel J, Winkler WC 2010 Identification of regulatory RNAs in *Bacillus subtilis*. Nucleic Acids Res 38:6637–6651. doi:10.1093/nar/gkq454. 20525796 PMC2965217

