# Supplementary figures and images for: In Vivo Behavior of the Tandem Glycine Riboswitch in Bacillus subtilis
Source: mBio. 2017 Oct 31;8(5):e01602-17. doi: 10.1128/mBio.01602-17 (PMC5666159; doi:10.1128/mBio.01602-17)

**A**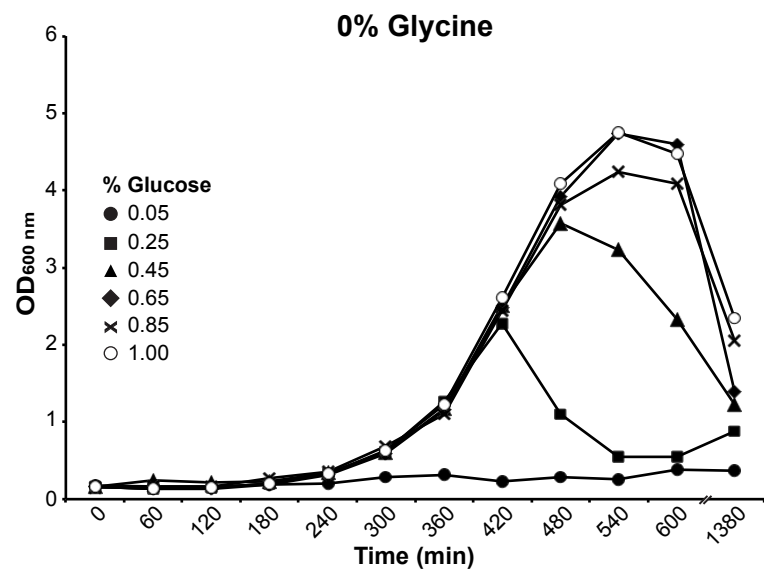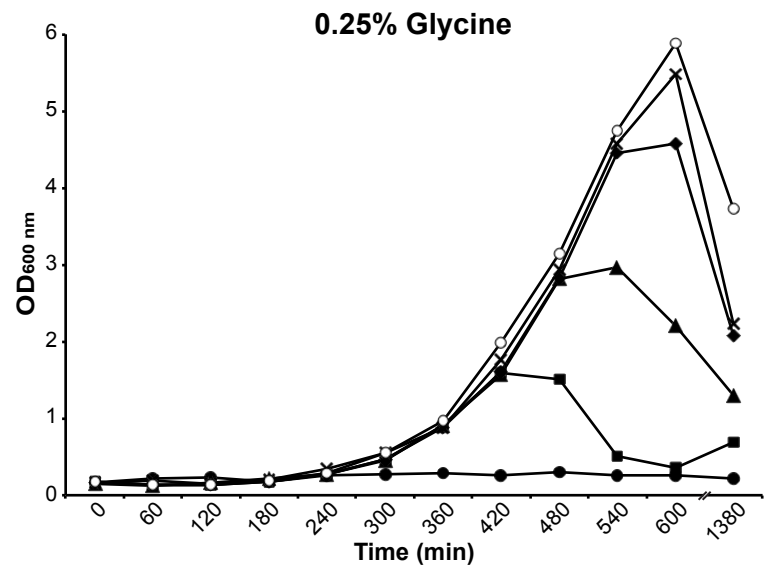**B**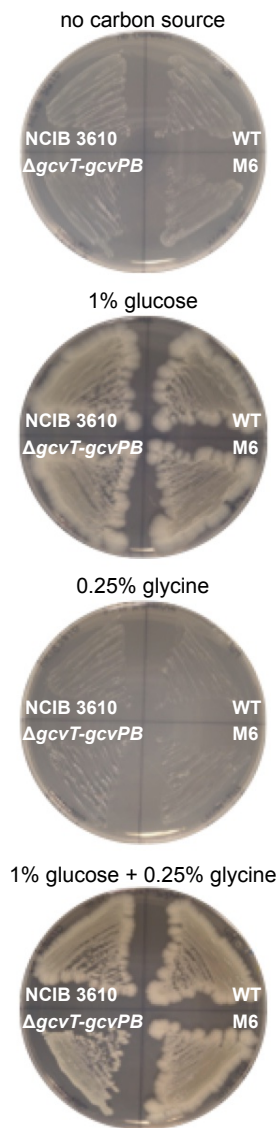

Supplement: FIG S1 [file mbo005173565sf1.pdf]
