# Supplementary material for: In Vivo Behavior of the Tandem Glycine Riboswitch in Bacillus subtilis
Source: mBio. 2017 Oct 31;8(5):e01602-17. doi: 10.1128/mBio.01602-17 (PMC5666159; doi:10.1128/mBio.01602-17)
Supplement: TABLE S1 [file mbo005173565st1.pdf]

Table S1. Oligonucleotides used in this study.

Name	Sequence (5'-3')	Notes
1018	GGGCCC**GAATTC**CTGTTGCCCAGCATATAGTGATGATGGTA	Primer for cloning glycine riboswitch into modified pDG1728 reporter vector; EcoRI restriction site in bold
1019	CCGGCC**GGATCC**GTCAAATAACGGCGTTCTTTTCAGCAT	Primer for cloning glycine riboswitch into modified pDG1728 reporter vector; BamHI restriction site in bold
204	TATCTCTTGCCAGTCACGTTACG	Primer for PCR checks and sequencing of pDG1728 reporter constructs
122	GGGGACGACGACAGTATCGGCCTC	Primer for PCR checks and sequencing of pDG1728 reporter constructs
1014	TGACAAGATCATATGGGATAGACAG	Primer for amplifying 5'-500 bp region of glycine riboswitch recombinant construct
1015	CTTTAGGGTTATCGAATTCGATAAGCTTCTACAATTTGGGCAGATTTTCTTATATTATTCATC	Primer for amplifying 5'-500 bp region of glycine riboswitch recombinant construct
1016	TAGCGCCTACGGGGAATTTGTATCGCGGCCGCCTGTTGCCCAGCATATAGTGATGATGGTAGG	Primer for amplifying 3'-500 bp region of glycine riboswitch recombinant construct
1017	TCGCTGTATATTGAGCACGGCCTGG	Primer for amplifying 3'-500 bp region of glycine riboswitch recombinant construct
681	TAGAAGCTTATCGAATTCGATAACCCTAAAG	Primer for amplifying erythromycin resistance cassette from pDG1663
682	GCGGCCGCGATACAAATTAAAAGTAGGCG	Primer for amplifying erythromycin resistance cassette from pDG1663
1046	CGAGCTTCCGGACAAATTCATAGTTC	Primer for confirming genomic integration of recombinant constructs
1047	GAAGCATTAATGACAAGCAGATAGCG	Primer for confirming genomic integration of recombinant constructs
745	GCAATGAAACACGCCAAAGTAAAC	Primer for PCR checks, sequencing recombinant glycine riboswitch constructs
1648	CTGCGGAGTGAATCTCACAGGCAAAAGAACTC	Mutagenesis primer for M1, glycine-binding mutation to first aptamer

1649	GAGTTCTTTTGCCTGTGAGATTCACTCCGCAG	Mutagenesis primer for M1, glycine-binding mutation to first aptamer
1650	GCAAAGTAAACTTACAGGTGCCAGGACAGAG	Mutagenesis primer for M2, glycine-binding mutation to second aptamer
1651	CTCTGTCCTGGCACCTGTAAGTTTACTTTGC	Mutagenesis primer for M2, glycine-binding mutation to second aptamer
1263	GCAAACTGCGGAGTGAACCTCTCAGGCAAAAGAAC	Mutagenesis primer for M3, dimerization mutation to first aptamer
1264	GTTCTTTTGCCTGAGAGGTTCACTCCGCAGTTTGC	Mutagenesis primer for M3, dimerization mutation to first aptamer
1265	GCGTATGCAAAGTAAGCTTTCAGGTGCCAGG	Mutagenesis primer for M4, dimerization mutation to second aptamer
1266	CCTGGCACCTGAAAGCTTACTTTGCATACGC	Mutagenesis primer for M4, dimerization mutation to second aptamer
1526	CATGAAAATATGAGCGAATCCCAGCAAGGGGAGAGAC	Mutagenesis primer for M5, leader-linker kink-turn mutation
1527	GTCTCTCCCCTTGCTGGGATTCGCTCATATTTTCATG	Mutagenesis primer for M5, leader-linker kink-turn mutation
1284	GGTGTTTCTCTGTAATTTTTTGTATG	Mutagenesis primer for M6, terminator mutation
1285	CATACAAAAAATTACAGAGAAACACC	Mutagenesis primer for M6, terminator mutation
1528	GACCTGACCGAAAATTTCGGGATACAGGCGC	Mutagenesis primer for M7, control mutation to first aptamer
1529	GCGCCTGTATCCCGAAATTTTCGGTCAGGTC	Mutagenesis primer for M7, control mutation to first aptamer
1530	GAGTGTTTGTGCGGAAGCGCAAACCACCAAAGG	Mutagenesis primer for M8, control mutation to second aptamer
1531	CCTTTGGTGGTTTGCGCTTCCGCACAAACACTC	Mutagenesis primer for M8, control mutation to second aptamer
1444	CTGACAGCTTCCAAGGAGCTAAAGAGGTCTCCTGTTGATAGATCCAGTAATGACC	Primer for amplifying double terminator construct from pYH213 for appending onto 3' end of erythromycin resistance cassette from pDG1663 for Δ*gcvT-gcvPB* recombinant construct

1445	GGTCATTACTGGATCTATCAACAGGAGACCTCTTTAGCTCCTTGGAAGCTGTCAG	Primer for amplifying double terminator construct from pYH213 for appending onto 3’ end of erythromycin resistance cassette from pDG1663 for Δ*gcvT-gcvPB* recombinant construct
1446	GTTTAAACGATACAAATTCCCCGTAGGCGCTAGGGAAAAAAATTACGCCCCGCCCTGCC	Primer for appending double terminator construct onto 3’ end of erythromycin resistance cassette from pDG1663 for Δ*gcvT-gcvPB* recombinant construct; use with primer 681
1652	CCCTAGCGCCTACGGGGAATTTGTATCGTTTAAACATAAAAACAGCTGTCTACCAGACAG	Primer for amplifying 3'-500 bp region of Δ*gcvT-gcvPB* recombinant construct
1653	CGAAAACGGCTCTATGACCTTG	Primer for amplifying 3'-500 bp region of Δ*gcvT-gcvPB* recombinant construct
1654	GAATCAGTTTATCAAACTGTCGGG	Primer for confirming genomic integration of Δ*gcvT-gcvPB* recombinant construct; use with primer 1046
1671	AAAGGAGAGAACCGCTATCTGC	Primer for qRT-PCR targeting the *gcvT* coding region
1672	AATCTGCACATCACCTGCTG	Primer for qRT-PCR targeting the *gcvT* coding region
1546	TTTTACTTCGTGACGGCGGT	Primer for qRT-PCR targeting *nifU*, the normalization control
1547	TTGTTGAACTTGGGCAGCTG	Primer for qRT-PCR targeting *nifU*, the normalization control
