# Supplementary material for: In Vivo Behavior of the Tandem Glycine Riboswitch in Bacillus subtilis
Source: mBio. 2017 Oct 31;8(5):e01602-17. doi: 10.1128/mBio.01602-17 (PMC5666159; doi:10.1128/mBio.01602-17)
Supplement: TEXT S1 [file mbo005173565s1.pdf]

SUPPLEMENTAL MATERIALS AND METHODS

Growth curves with varying glucose concentrations. *B. subtilis* NCIB 3610 starter cultures were prepared as described and used to inoculate 0.5 mL M9 minimal medium +/- 0.25% glycine cultures with varying glucose concentrations (0%, 0.2%, 0.4%, 0.6%, 0.8%, and 1% w/v glucose) in sterile non-treated 24-well cell culture plates to a starting OD_{600} reading of approximately 0.2. Plates were incubated at 37ºC with shaking (225 rpm) for approximately 24 hours. OD_{600} values were recorded at time points indicated using a SpectraMax M3 Multi-Mode Microplate Reader (Molecular Devices). For further analysis, select *B. subtilis* NCIB 3610 strains were patched onto the following plates (1.5% agar): M9 minimal medium + no carbon source, M9 minimal medium + 1% glucose, M9 minimal medium + 0.25% glycine, and M9 minimal medium + 1% glucose + 0.25% glycine. Plates were incubated at 37ºC for one week and photographs were taken using a Samsung WB380F digital camera.
